# Characteristics of Deceased Solid Organ Donors and Screening Results for Hepatitis B, C, and Human Immunodeficiency Viruses — United States, 2010–2017

**DOI:** 10.15585/mmwr.mm6803a2

**Published:** 2019-01-25

**Authors:** Winston E. Abara, Melissa G. Collier, Anne Moorman, Danae Bixler, Jefferson Jones, Pallavi Annambhotla, James Bowman, Marilyn E. Levi, John T. Brooks, Sridhar V. Basavaraju

**Affiliations:** ^1^Division of Viral Hepatitis, National Center for HIV/AIDS, Viral Hepatitis, STD, and TB, CDC; ^2^Division of Healthcare Quality Promotion, National Center for Emerging and Zoonotic Infectious Diseases, CDC; ^3^Health Resources and Services Administration, Rockville, Maryland; ^4^Division of HIV/AIDS Prevention, National Center for HIV/AIDS, Viral Hepatitis, STD, and TB, CDC.

The ongoing U.S. opioid crisis has resulted in an increase in drug overdose deaths and acute hepatitis C virus (HCV) infections, with young persons (who might be eligible organ donors) most affected.*^,^^†^ In 2013, the Public Health Service released a revised guideline to reduce the risk for unintended organ transplantation–associated hepatitis B virus (HBV), HCV, and human immunodeficiency virus (HIV) transmission (*1*). The guideline describes criteria to categorize donors at increased risk (increased risk donors [IRDs]) for transmitting these viruses to recipients (*1*). It also recommends universal donor testing for HBV, HCV, and HIV.^§^ CDC analyzed deceased donor data for the period 2010–2017 reported to the Organ Procurement and Transplantation Network for IRDs and standard risk donors (SRDs) (i.e., donors who do not meet any of the criteria for increased risk designation). During this period, the proportion of IRDs increased approximately 200%, from 8.9% to 26.3%; the percentage with drug intoxication reported as the mechanism of death also increased approximately 200%, from 4.3% to 13.4%; and the proportion of these donors with reported injection drug use (IDU) increased approximately 500%, from 1.3% to 8.0%. Compared with SRDs, IRDs were significantly more likely to have positive HBV and HCV screening results. These findings demonstrate the continuing need for identifying viral bloodborne pathogen infection risk factors among deceased donors to reduce the risk for transmission, monitor posttransplant infection in recipients, and offer treatment if infection occurs.

In the United States, all organ procurement organizations and transplant centers participate in the Organ Procurement and Transplantation Network, which is operated by the United Network for Organ Sharing through a contract with the Health Resources and Services Administration (HRSA). Participating facilities report donor data to the United Network for Organ Sharing, including donor risk type (i.e., increased or standard risk), age, sex, race, mechanism of death (further stratified by drug intoxication and history of IDU), and HBV, HCV, and HIV screening results.^¶^ Data for all deceased solid organ donors with one or more organs recovered for the purpose of transplantation during January 1, 2010–December 31, 2017 were analyzed.

Descriptive statistics and frequencies were calculated by year to assess trends in demographic characteristics and HBV, HCV, and HIV screening results among all donors and by donor risk type. The change in the proportions of IRDs, SRDs, drug intoxication reported as mechanism of death, and IDU history from 2010 to 2017 along with comparisons of HBV and HCV screening results between IRDs and SRDs were assessed using the chi-squared test, with p-values <0.05 considered statistically significant. Anti-HCV and anti-HIV screening results for the period 2010–2017, and nucleic acid test (NAT) results for the period 2014–2017 were used because implementation of the guideline recommendation for HCV and HIV testing by NAT did not begin until 2014.** Statistical software was used to conduct all analyses.

## Deceased Donors

The annual number of deceased donors increased 29.5%, from 7,943 in 2010 to 10,287 in 2017 (Table 1). Among the 70,414 deceased donors during this period, 57,782 (82.1%), 12,592 (17.9%), and 40 (<0.1%) were classified as SRDs, IRDs, and unknown risk, respectively. The mean donor age was 39.9 years, 59.6% were male, and 66.2% were white. The number of deceased donors with drug intoxication reported as the mechanism of death increased from 342 (4.3%) in 2010 to 1,382 (13.4%) in 2017 (p<0.001). Among those with drug intoxication as mechanism of death, the number with IDU history increased from 107 (1.3%) in 2010 to 825 (8.0%) in 2017 (p<0.001). From 2010 to 2017, hepatitis B surface antigen (HBsAg) positivity remained constant (0.1%), total hepatitis B core antibody (anti-HBc) positivity (indicating previous or ongoing HBV infection) decreased slightly (from 5.0% to 4.7%), anti-HCV positivity increased (4.2% to 7.3%), and anti-HIV positivity increased slightly (0.0% to 0.1%). From 2014 to 2017, HCV RNA positivity increased (3.9% to 4.9%).

**TABLE 1:** Characteristics of deceased organ donors (N = 70,414) — Organ Procurement and Transplantation Network, United States, 2010–2017

Characteristic	2010	2011	2012	2013	2014	2015	2016	2017	Total 2010–2017
	No. (%)	No. (%)	No. (%)	No. (%)	No. (%)	No. (%)	No. (%)	No. (%)	No. (%)
**Total**	**7,943**	**8,126**	**8,143**	**8,269**	**8,596**	**9,079**	**9,971**	**10,287**	**70,414 (100)**
Risk type for deceased donor*									
Standard risk	7,226 (91.0)	7,283 (89.6)	7,171 (88.1)	7,157 (86.6)	6,815 (79.3)	7,059 (77.8)	7,491 (75.1)	7,580 (73.7)	**57,782 (82.1)**
Increased risk	709 (8.9)	836 (10.3)	966 (11.9)	1,111 (13.4)	1,772 (20.6)	2,016 (22.2)	2,478 (24.9)	2,704 (26.3)	**12,592 (17.9)**
**Mean age (yrs), (SD)**	40.5 (18.2)	40.1 (18.1)	39.8 (18.0)	40.1 (18.0)	40.1 (17.6)	39.5 (17.9)	39.5 (17.3)	40.0 (17.1)	**39.9 (17.8)**
**Age group (yrs)**									
0–17	841 (10.6)	881 (10.8)	852 (10.5)	873 (10.6)	841 (9.8)	939 (10.3)	934 (9.4)	896 (8.7)	**7,057 (10.0)**
18–24	1,053 (13.3)	1,060 (13.0)	1,095 (13.5)	1,041 (12.6)	1,079 (12.6)	1,218 (13.4)	1,220 (12.2)	1,210 (11.8)	**8,976 (12.8)**
25–34	1,116 (14.1)	1,181 (14.5)	1,240 (15.2)	1,278 (15.5)	1,395 (16.2)	1,490 (16.4)	1,885 (18.9)	1,962 (19.1)	**11,547 (16.4)**
35–44	1,196 (15.1)	1,247 (15.4)	1,209 (14.9)	1,335 (16.1)	1,380 (16.1)	1,473 (16.2)	1,708 (17.1)	1,766 (17.2)	**11,314 (16.0)**
45–54	1,770 (22.3)	1,808 (22.3)	1,870 (23.0)	1,782 (21.6)	1,888 (22.0)	1,869 (20.6)	2,006 (20.1)	2,063 (20.0)	**15,056 (21.4)**
55–64	1,298 (16.3)	1,354 (16.7)	1,303 (16.0)	1,326 (16.0)	1,399 (16.3)	1,472 (16.2)	1,590 (16.0)	1,724 (16.8)	**11,466 (16.3)**
≥65	669 (8.4)	595 (7.3)	574 (7.1)	634 (7.7)	614 (7.1)	618 (6.8)	628 (6.3)	666 (6.4)	**4,998 (7.1)**
**Sex**									
Male	4,683 (59.0)	4,764 (58.6)	4,820 (59.2)	4,906 (59.3)	5,164 (60.1)	5,486 (60.4)	5,957 (59.7)	6,200 (60.3)	**41,980 (59.6)**
Female	3,260 (41.0)	3,362 (41.4)	3,323 (40.8)	3,363 (40.7)	3,432 (39.9)	3,593 (39.6)	4,014 (40.3)	4,087 (39.7)	**28,434 (40.4)**
**Race**									
White	5,284 (66.5)	5,397 (66.4)	5,382 (66.1)	5,461 (66.0)	5,709 (66.4)	5,966 (65.7)	6,647 (66.7)	6,790 (66.0)	**46,636 (66.2)**
Black	1,323 (16.6)	1,296 (16.0)	1,369 (16.8)	1,371 (16.6)	1,341 (15.6)	1,476 (16.3)	1,569 (15.7)	1,603 (15.6)	**11,348 (16.1)**
Hispanic	1,029 (13.0)	1,078 (13.2)	1,033 (12.7)	1,111 (13.5)	1,144 (13.3)	1,236 (13.6)	1,310 (13.1)	1,434 (13.9)	**9,375 (13.3)**
Other**^†^**	307 (3.9)	355 (4.4)	359 (4.4)	326 (3.9)	402 (4.7)	401 (4.4)	445 (4.5)	460 (4.5)	**3,055 (4.4)**
**Mechanism of death**									
Drug intoxication	342 (4.3)	473 (5.8)	440 (5.4)	560 (6.8)	625 (7.3)	848 (9.3)	1,262 (12.7)	1,382 (13.4)	**5,932 (8.4)**
Injection drug use^§^	107 (1.3)	169 (2.1)	178 (2.2)	248 (3.0)	332 (3.9)	471 (5.2)	727 (7.3)	825 (8.0)	**3,057 (4.3)**
**Hepatitis B surface antigen**									
Positive	7 (0.1)	6 (0.1)	6 (0.1)	7 (0.1)	7 (0.1)	8 (0.1)	9 (0.1)	11 (0.1)	**61 (0.1)**
Negative	7,934 (99.9)	8,120 (99.9)	8,137 (99.9)	8,261 (99.9)	8,588 (99.9)	9,071 (99.9)	9,962 (99.9)	10,276 (99.9)	**70,349 (99.9)**
**Hepatitis B core antibody**									
Positive	398 (5.0)	369 (4.5)	400 (4.9)	382 (4.6)	419 (4.9)	440 (4.9)	498 (5.0)	484 (4.7)	**3,390 (4.8)**
Negative	7,541 (95.0)	7,755 (95.5)	7,741 (95.1)	7,883 (95.4)	8,176 (95.1)	8,639 (95.1)	9,473 (95.0)	9,803 (95.3)	**67,011 (95.2)**
**HCV antibody**									
Positive	331 (4.2)	320 (3.9)	335 (4.1)	361 (4.4)	436 (5.1)	535 (5.9)	661 (6.6)	746 (7.3)	**3,725 (5.3)**
Negative	7,609 (95.2)	7,806 (96.1)	7,806 (95.9)	7,908 (95.6)	8,160 (94.1)	8,544 (94.1)	9,309 (93.4)	9,541 (92.7)	**66,683 (94.7)**
**HCV RNA by NAT**									
Positive	—	—	—	—	12 (3.9)	330 (4.2)	461 (4.6)	503 (4.9)	**1,306 (4.6)**
Negative	—	—	—	—	298 (96.1)	7,482 (95.8)	9,509 (95.4)	9,783 (95.1)	**27,072 (95.4)**
**HIV antibody^¶^**									
Positive	0 (0.0)	0 (0.0)	0 (0.0)	0 (0.0)	1 (0.0)	2 (0.0)	7 (0.1)	13 (0.1)	**23 (0.0)**
Negative	7,936 (100.0)	8,123 (100.0)	8,140 (100.0)	8,265 (100.0)	8,539 (100.0)	8,821 (100.0)	9,742 (99.9)	10,206 (99.9)	**69,772 (100.0)**

**Abbreviations**: HCV = hepatitis C virus; HIV = human immunodeficiency virus; NAT = nucleic acid test; SD = standard deviation.

* 40 deceased donors were categorized with unknown risk type.

**^†^** Includes Asian, America Indian/Alaska Native, Native Hawaiian, and multiracial.

**^§^** Among those with drug intoxication reported as a mechanism of death and a reported history of injection drug use.

^¶^ The HIV Organ Policy Equity Act (HOPE Act) of 2013 allows transplantation, under research protocols, of organs from donors infected with HIV into recipients who are also infected with HIV. https://optn.transplant.hrsa.gov/governance/public-comment/changes-to-hope-act-open-variance/.

## Increased Risk Donors

The number and percentage of IRDs among all deceased donors increased from 709 (8.9%) in 2010 to 2,704 (26.3%) in 2017 (Table 2) (p<0.001). Among IRDs, mean age was 35.2 years, 66.3% were male, and 70.0% were white. From 2010 to 2017, there were no substantial changes in HBsAg or anti-HBc positivity; however, anti-HCV positivity increased (15.9% to 21.6%). From 2014 to 2017, HCV RNA positivity also increased (8.6% to 15.7%).

**TABLE 2:** Characteristics of deceased increased risk donors (IRDs) (N = 12,592) — Organ Procurement and Transplantation Network, United States, 2010–2017

Characteristic	2010	2011	2012	2013	2014	2015	2016	2017	Total 2010–2017
	No. (%)	No. (%)	No. (%)	No. (%)	No. (%)	No. (%)	No. (%)	No. (%)	No. (%)
**IRDs (% among all deceased donors)**	**709 (8.9)**	**836 (10.3)**	**966 (11.9)**	**1,111 (13.4)**	**1,772 (20.6)**	**2,016 (22.2)**	**2,478 (24.9)**	**2,704 (26.3)**	**12,592 (17.9)**
**Mean age (yrs), (SD)**	34.8 (14.3)	34.5 (14.1)	34.0 (14.5)	34.3 (14.1)	35.5 (14.1)	35.2 (13.7)	35.4 (13.2)	35.9 (13.1)	**35.2 (13.7)**
**Age group (yrs)**									
0–17	45 (6.4)	37 (4.4)	71 (7.4)	72 (6.5)	96 (5.4)	98 (4.7)	93 (3.8)	103 (3.8)	**615 (4.8)**
18–24	150 (21.2)	195 (23.3)	200 (20.7)	228 (20.5)	337 (19.0)	382 (19.0)	405 (16.3)	394 (14.6)	**2,291 (18.2)**
25–34	187 (26.4)	241 (28.8)	284 (29.4)	307 (27.6)	504 (28.4)	610 (30.3)	840 (33.9)	899 (33.3)	**3,872 (30.8)**
35–44	127 (17.9)	146 (17.5)	159 (16.5)	230 (20.7)	337 (19.0)	411 (20.4)	520 (21.0)	618 (22.8)	**2,548 (20.2)**
45–54	130 (18.3)	133 (15.9)	166 (17.2)	174 (15.7)	302 (17.0)	311 (15.4)	387 (15.6)	411 (15.2)	**2,014 (16.0)**
55–64	54 (7.6)	69 (8.3)	65 (6.7)	83 (7.5)	155 (8.8)	158 (7.8)	181 (7.3)	223 (8.2)	**988 (7.9)**
≥65	16 (2.3)	15 (1.8)	21 (2.2)	17 (1.5)	41 (2.3)	46 (2.3)	52 (2.1)	56 (2.1)	**264 (2.1)**
**Sex**									
Male	476 (67.1)	552 (66.0)	642 (66.5)	737 (66.3)	1,184 (66.8)	1,360 (67.5)	1,637 (66.1)	1,760 (65.1)	**8,348 (66.3)**
Female	233 (32.9)	284 (34.0)	324 (33.5)	374 (33.7)	588 (33.2)	656 (32.5)	841 (33.9)	944 (34.9)	**4,244 (33.7)**
**Race**									
White	529 (74.6)	617 (73.8)	728 (75.4)	804 (72.4)	1,191 (67.2)	1,366 (67.8)	1,734 (70.0)	1,849 (68.4)	**8,818 (70.0)**
Black	101 (14.2)	107 (12.8)	131 (13.6)	152 (13.7)	296 (16.7)	334 (16.6)	363 (14.7)	431 (15.9)	**1,915 (15.2)**
Hispanic	68 (9.6)	101 (12.1)	88 (9.1)	137 (12.3)	222 (12.5)	252 (12.5)	302 (12.2)	335 (12.4)	**1,505 (12.0)**
Other*	11 (1.6)	11 (1.3)	19 (1.9)	18 (1.6)	63 (3.6)	64 (3.1)	79 (3.1)	89 (3.3)	**354 (2.8)**
**Hepatitis B surface antigen**									
Positive	0 (0)	0 (0)	0 (0)	0 (0)	2 (0.1)	5 (0.3)	4 (0.2)	3 (0.1)	**14 (0.1)**
Negative	709 (100.0)	836 (100.0)	966 (100.0)	1,111 (100.0)	1,770 (99.9)	2,011 (99.7)	2,474 (99.8)	2,701 (99.9)	**12,578 (99.9)**
**Hepatitis B core antibody**									
Positive	57 (8.0)	51 (6.1)	77 (8.0)	79 (7.1)	134 (7.6)	126 (6.3)	173 (7.0)	189 (7.0)	**886 (7.0)**
Negative	652 (92.0)	784 (93.9)	889 (92.0)	1,032 (92.9)	1,638 (92.4)	1,890 (93.7)	2,305 (93.0)	2,515 (93.0)	**11,705 (93.0)**
**HCV antibody**									
Positive	113 (15.9)	137 (16.4)	154 (15.9)	201 (18.1)	313 (17.7)	390 (19.4)	509 (20.5)	583 (21.6)	**2,400 (19.1)**
Negative	596 (84.1)	699 (83.6)	812 (84.1)	910 (81.9)	1,459 (82.3)	1,626 (80.7)	1,969 (79.5)	2,121 (78.4)	**10,192 (80.9)**
**HCV RNA by NAT**									
Positive	—	—	—	—	7 (8.6)^†^	252 (14.5)^§^	363 (14.7)^¶^	423 (15.7)**	**1,045 (14.9)**
Negative	—	—	—	—	74 (91.4)	1,488 (85.5)	2,114 (85.3)	2,280 (84.3)	**5,956 (85.1)**
**Percentage of IRDs tested for HCV RNA by NAT**	—	—	—	—	81 (4.6)	1,740 (86.3)	2,477 (>99.9)	2,703 (>99.9)	**7,001 (78.1)**
**HIV antibody^††^**									
Positive	0 (0.0)	0 (0.0)	0 (0.0)	0 (0.0)	0 (0.0)	0 (0.0)	3 (0.1)	7 (0.3)	**10 (0.1)**
Negative	708 (100.0)	836 (100.0)	966 (100.0)	1,111 (100.0)	1,762 (100.0)	1,969 (100.0)	2,410 (99.9)	2,667 (99.7)	**12,429 (99.9)**
**HIV RNA by NAT**									
Positive	—	—	—	—	0 (0.0)	0 (0.0)	2 (0.1)	4 (0.2)	**6 (0.1)^§§^**
Negative	—	—	—	—	79 (100.0)	1,733 (100.0)	2,468 (99.9)	2,698 (99.8)	**6,978 (99.9)**
**Percentage of IRDs tested for HIV RNA by NAT**	—	—	—	—	79 (4.5)	1,733 (86.0)	2,470 (99.7)	2,702 (99.9)	**6,984 (77.9)**
**HIV p24 antigen**									
Positive	—	—	—	—	0 (0.0)	0 (0.0)	0 (0.0)	0 (0.0)	**0 (0.0)**
Negative	—	—	—	—	2 (100.0)	59 (100.0)	78 (100.0)	36 (100.0)	**175 (100.0)**
**HBV DNA by NAT**									
Positive	—	—	—	—	0 (0.0)	8 (0.5)	9 (0.4)	9 (0.3)	**26 (0.4)**
Negative	—	—	—	—	81 (100.0)	1,732 (99.5)	2,467 (99.6)	2,694 (99.7)	**6,974 (99.6)**
**Percentage of IRDs tested for HBV DNA by NAT**	—	—	—	—	81 (4.6)	1,740 (86.3)	2,467 (99.6)	2,703 (>99.9)	**6,991 (77.9)**

**Abbreviations:** HBV = hepatitis B virus; HCV = hepatitis C virus; HIV = human immunodeficiency virus; NAT = nucleic acid test; SD = standard deviation.

* Includes Asian, America Indian/Alaska Native, Native Hawaiian, and multiracial.

**^†^** Six of the seven HCV RNA–positive donors were anti-HCV positive; one was negative.

**^§^** 243 of 252 (96.4%) HCV RNA–positive donors were anti-HCV positive; nine (3.6%)were negative.

^¶^ 344 of 363 (94.8%) HCV RNA–positive donors were anti-HCV positive; 19 (5.2%) were negative.

** 397 of 423 (93.9%) HCV RNA–positive donors were anti-HCV positive; 26 (6.1%) were negative.

^††^ The HIV Organ Policy Equity Act (HOPE Act) of 2013 allows transplantation, under research protocols, of organs from donors infected with HIV into recipients who are also infected with HIV. https://optn.transplant.hrsa.gov/governance/public-comment/changes-to-hope-act-open-variance/.

^§§^ Five of the six HIV RNA-positive donors were anti-HIV positive; one (16.7%) was negative.

From 2014 to 2017, the percentage of IRDs tested by HCV and HIV NAT increased from 4.6% to >99.9% and from 4.5% to 99.9%, respectively. During this period, 55 (one in 2014; nine in 2015; 19 in 2016; and 26 in 2017) or 5.3% of all HCV RNA–positive IRDs were anti-HCV negative (i.e., acute infection before antibody response). From 2014 to 2017, the percentage of IRDs tested by HBV NAT increased from 4.6% to >99.9%.

## Standard Risk Donors

Whereas the number of deceased SRDs rose from 7,226 in 2010 to 7,580 in 2017, the percentage of SRDs among all deceased donors decreased from 90.1% to 73.7% (Table 3) (p<0.001). Among SRDs, the mean age was 41.0 years, 58.2% were male, and 65.4% were white. From 2010 to 2017, HBsAg positivity remained constant (0.1%), whereas anti-HBc and anti-HCV positivity decreased (from 4.7% to 3.9% and from 3.0% to 2.2%, respectively). From 2014 to 2017, HCV RNA positivity decreased from 2.2% to 1.1%.

**TABLE 3:** Characteristics of deceased standard risk donors (SRDs) (N = 57,782) — Organ Procurement and Transplantation Network, United States, 2010–2017

Characteristic	2010	2011	2012	2013	2014	2015	2016	2017	Total
	No. (%)	No. (%)	No. (%)	No. (%)	No. (%)	No. (%)	No. (%)	No. (%)	No. (%)
**SRDs (% of all deceased donors)**	**7,226 (90.1)**	**7,283 (89.6)**	**7,171 (88.1)**	**7,157 (86.6)**	**6,815 (79.3)**	**7,059 (77.8)**	**7,491 (75.1)**	**7,580 (73.7)**	**57,782 (82.1)**
**Mean age (yrs), (SD)**	41.0 (18.5)	40.7 (18.4)	40.6 (18.3)	41.0 (18.4)	41.3 (18.3)	40.7 (18.8)	40.8 (18.2)	41.4 (18.1)	**41.0 (18.4)**
**Age group (yrs)**									
0–17	795 (11.0)	844 (11.6)	781 (10.9)	801 (11.2)	745 (10.9)	839 (11.9)	841 (11.2)	792 (10.5)	**6,438 (11.1)**
18–24	902 (12.5)	865 (11.9)	895 (12.5)	813 (11.4)	741 (10.9)	836 (11.8)	814 (10.9)	816 (10.7)	**6,682 (11.6)**
25–34	929 (12.9)	940 (12.9)	955 (13.3)	971 (13.6)	891 (13.1)	880 (12.5)	1,044 (13.9)	1,063 (14.0)	**7,673 (13.3)**
35–44	1,067 (14.8)	1,101 (15.1)	1,049 (14.6)	1,104 (15.4)	1,041 (15.3)	1,062 (15.0)	1,188 (15.9)	1,147 (15.1)	**8,759 (15.2)**
45–54	1,639 (22.7)	1,672 (23.0)	1,702 (23.7)	1,608 (22.5)	1,582 (23.2)	1,557 (22.1)	1,619 (21.6)	1,651 (21.8)	**13,030 (22.6)**
55–64	1,242 (17.2)	1,283 (17.6)	1,238 (17.3)	1,243 (17.4)	1,243 (18.2)	1,314 (18.6)	1,409 (18.8)	1,501 (19.8)	**10,473 (18.1)**
≥65	652 (9.0)	578 (7.9)	551 (7.7)	617 (8.6)	572 (8.4)	571 (8.1)	576 (7.7)	610 (8.1)	**4,727 (8.1)**
**Sex**									
Male	4,202 (58.2)	4,207 (57.8)	4,175 (58.2)	4,169 (58.3)	3,973 (58.3)	4,124 (58.4)	4,320 (57.7)	4,437 (58.5)	**33,607 (58.2)**
Female	3,024 (41.9)	3,076 (42.2)	2,996 (41.8)	2,988 (41.8)	2,842 (41.7)	2,935 (41.6)	3,171 (42.3)	3,143 (41.5)	**24,175 (41.8)**
**Race**									
White	4,751 (65.7)	4,776 (65.6)	4,651 (64.9)	4,657 (65.1)	4,515 (66.2)	4,599 (65.2)	4,912 (65.5)	4,941 (65.2)	**37,802 (65.4)**
Black	1,220 (16.9)	1,188 (16.3)	1,235 (17.2)	1,218 (17.0)	1,043 (15.3)	1,140 (16.1)	1,205 (16.1)	1,170 (15.4)	**9,419 (16.3)**
Hispanic	959 (13.3)	975 (13.4)	945 (13.2)	974 (13.6)	919 (13.5)	983 (13.9)	1,008 (13.5)	1,098 (14.5)	**7,861 (13.6)**
Other*	296 (4.1)	344 (4.7)	340 (4.7)	308 (4.3)	338 (5.0)	337 (4.8)	366 (4.9)	371 (4.9)	**2,700 (4.7)**
**Hepatitis B surface antigen**									
Positive	7 (0.1)	6 (0.1)	6 (0.1)	7 (0.1)	5 (0.1)	3 (0.1)	5 (0.1)	8 (0.1)	**47 (0.1)**
Negative	7,217 (99.9)	7,277 (99.9)	7,165 (99.9)	7,149 (99.9)	6,809 (99.9)	7,056 (99.9)	7,486 (99.9)	7,572 (99.9)	**57,731 (99.9)**
**Hepatitis B core antibody**									
Positive	340 (4.7)	318 (4.4)	321 (4.5)	303 (4.2)	285 (4.2)	314 (4.5)	325 (4.3)	295 (3.9)	**2,501 (4.3)**
Negative	6,882 (95.3)	6,964 (95.6)	6,848 (95.5)	6,850 (95.8)	6,529 (95.8)	6,745 (95.5)	7,166 (95.7)	7,285 (96.1)	**55,269 (95.7)**
**HCV antibody**									
Positive	217 (3.0)	182 (2.5)	181 (2.5)	160 (2.2)	123 (1.8)	145 (2.1)	152 (2.0)	163 (2.2)	**1,323 (2.3)**
Negative	7,006 (97.0)	7,101 (97.5)	6,988 (97.5)	6,997 (97.8)	6,692 (98.2)	6,914 (97.9)	7,338 (98.0)	7,417 (97.8)	**56,453 (97.7)**
**HCV RNA by NAT**									
Positive	—	—	—	—	5 (2.2)**^†^**	78 (1.3)**^§^**	98 (1.3)^¶^	80 (1.1)**	**261 (1.2)**
Negative	—	—	—	—	224 (97.8)	5,991 (98.7)	7,393 (98.7)	7,500 (98.9)	**21,108 (98.8)**
**SRDs tested for HCV RNA by NAT**	—	—	—	—	229 (3.4)	6,069 (86.0)	7,491 (100.0)	7,580 (100.0)	**21,369 (73.8)**
**HIV antibody^††^**									
Positive	0 (0.0)	0 (0.0)	0 (0.0)	0 (0.0)	1 (0.0)	2 (0.0)	4 (0.1)	6 (0.1)	**13 (0.0)**
Negative	7,220 (100.0)	7,280 (100.0)	7,168 (100.0)	7,153 (100.0)	6,768 (100.0)	6,848 (100.0)	7,331 (99.9)	7,536 (99.9)	**57,304 (100.0)**
**HIV RNA by NAT**									
Positive	—	—	—	—	0 (0.0)	1 (0.0)	1 (0.0)	2 (0.0)	**4 (0.0)**
Negative	—	—	—	—	225 (100.0)	5,951 (100.0)	7,407 (100.0)	7,578 (100.0)	**21,161 (100.0)**
**SRDs tested for HIV RNA by NAT**	—	—	—	—	225 (3.3)	5,952 (84.3)	7,408 (98.9)	7,580 (100.0)	**21,165 (73.1)**
**HBV DNA by NAT**									
Positive	—	—	—	—	1 (0.4)	11 (0.2)	11 (0.1)	7 (0.1)	**30 (0.1)**
Negative	—	—	—	—	227 (99.6)	6,060 (99.8)	7,480 (99.9)	7,573 (99.9)	**21,340 (99.9)**
**SRDs tested for HBV DNA by NAT**	—	—	—	—	228 (3.3)	6,071 (86.0)	7,491 (100.0)	7,580 (100.0)	**21,370 (73.8)**

**Abbreviations**: HBV = hepatitis B virus; ; HCV = hepatitis C virus; HIV = human immunodeficiency virus; NAT = nucleic acid test; SD = standard deviation.

* Other include Asian, America Indian/Alaska Native, Native Hawaiian, Multiracial.

**^†^** All five (100%) HCV RNA–positive donors were anti-HCV positive.

**^§^** 74 of 78 (94.9%) HCV RNA–positive donors were anti-HCV positive; 4 (5.1%) were negative.

^¶^ 96 of 98 (98.0%) HCV RNA–positive donors were anti-HCV positive; 2 (2.0%) were negative.

** 77 of 80 (96.3%) HCV RNA–positive donors were anti-HCV positive; 3 (3.8%) were negative.

^††^ The HIV Organ Policy Equity Act (HOPE Act) of 2013 allows transplantation, under research protocols, of organs from donors infected with HIV into recipients who are also infected with HIV. https://optn.transplant.hrsa.gov/governance/public-comment/changes-to-hope-act-open-variance/.

During 2014–2017, the percentage of SRDs tested by HCV NAT increased from 3.4% to 100.0%. During this period, among all HCV RNA–positive donors, nine (3.5%) were anti-HCV negative (four in 2015, two in 2016, and three in 2017). Although HIV NAT and HBV NAT are not recommended for SRDs, the percentage of SRDs tested for HIV and HBV by NAT increased from 3.3% to 100.0%. Compared with SRDs, IRDs were significantly more likely to be anti-HBc–positive (7.0% versus 4.3%, p<0.001), HBV DNA–positive (0.4% versus 0.1%, p<0.001), anti-HCV–positive (19.1% versus 2.3%, p<0.001), and HCV RNA–positive (14.9% versus 1.2%, p<0.001).

## Discussion

During 2010–2017, the number and percentage of IRDs among all deceased donors increased. Similar to persons who die from opioid overdose in the United States, IRDs were more frequently white, male, and aged 25–34 years (*2*). Compared with SRDs, IRDs had higher HCV prevalence as well as a higher number and prevalence of acute HCV infections. Increases in opioid overdose deaths have likely contributed to the increasing number and percentage of IRDs in the United States as reflected by the increase in drug intoxication as mechanism of death among donors.

Some reports suggest underuse of IRD organs (*3*). According to the current guideline, donors are categorized as IRDs if risk behaviors occurred within the 12 months preceding donation (*1*). Use of NAT has greatly reduced the window period of undetectable infection to, on average, 3–5 days for HCV, 11–13 days for HIV, and 20–22 days for HBV (*4*,*5*). Because universal donor NAT testing has been implemented since 2014, reduction of the 12-month period for IRD designation to a shorter interval warrants further consideration. Although this study does not assess the use of donor organs, modifications to current recommendations might increase their use while still protecting recipient safety. These modifications include shortening the 12-month interval to reduce the proportion of donors categorized as IRDs and reassessment of terminology that might currently be contributing to underuse of these organs.

Because of the increased risk for transmission of HBV, HCV, and HIV through transplantation of IRD organs, the guideline recommends posttransplant HBV, HCV, and HIV testing of IRD organ recipients, in addition to donor testing (*1*). Standard posttransplant recipient testing is not otherwise routinely performed. The prevalence of HCV RNA positivity among IRDs (14.9%) was more than 12 times that among SRDs (1.2%). Because IRDs are at higher risk for HCV infection, identifying donor infection risk factors and conveying this information to recipients and their clinicians is important. This might ensure that recipients are screened posttransplant and, if HCV infection is diagnosed, offered treatment. HIV transmission from deceased organ donors to transplant recipients has not been identified in the United States since 2007 (*6*). However, window period HCV transmissions from IRDs have been reported (*7*), and the investigation by CDC of additional donor-derived HBV or HCV transmissions is ongoing. Available data indicate direct-acting antiviral treatment might be safe and effective for transplant recipients with donor-derived HCV infection (*8*). Effective therapy is also available for HIV and HBV donor-derived infection (*9*,*10*).

The findings in this report are subject to at least five limitations. First, these analyses focused on donor characteristics and did not compare SRD and IRD recipient outcomes. Second, data are limited to donors from whom at least one organ was recovered and do not include persons who might have been considered for donation but from whom no organs were recovered. Therefore, the testing results and mechanism of death might not fully reflect all persons considered for organ donation. Third, because IRD status is often determined by interviews of next of kin who might not be fully aware of donor risk behaviors, misclassification bias is possible. Fourth, the HIV Organ Policy Equity (HOPE) Act^††^ of 2013 permits the transplantation, under research protocols, of organs from donors with HIV infection to recipients who also have HIV infection. Data were unavailable to determine whether HIV antibody–positive or HIV RNA–positive donations occurred as part of the HOPE Act research studies, but these donors are likely to have been part of research studies covered by the HOPE Act, because donation from organ donors with diagnosed HIV infection is otherwise not permissible in the United States. Finally, the criteria for IRD designation changed with the 2013 revised guideline (*1*) and might have contributed to the observed increase in IRDs.

An increasing number of organ donors have a history of drug intoxication as the mechanism of death, mirroring the U.S. opioid crisis. These organ donors have high prevalence of HCV infection, but low prevalence of HIV and HBV infections. Identification of risk factors for viral bloodborne pathogen infection among organ donors is nonetheless important so that recipients and their clinicians can be notified and patients can be appropriately screened posttransplant. Prompt identification of posttransplant infection can facilitate early treatment. Given advances in technology and universal NAT implementation among solid organ donors, CDC and HRSA will continue to work with partners to review the current guideline recommendations to assess opportunities for refinement to reduce transmission of viral bloodborne pathogens and increase donor organ use.

**Summary**

**What is already known about this topic?**

Drug overdose deaths and hepatitis C virus (HCV) infections have increased with the U.S. opioid crisis. The Public Health Service guideline for reducing unintended organ transplantation–associated hepatitis B virus (HBV), HCV, and human immunodeficiency virus (HIV) transmission describes criteria to identify increased risk donors (IRDs).

**What is added by this report?**

The number and proportion of IRDs have increased since 2010, likely because of the epidemic of opioid overdose deaths. Compared with standard risk donors, IRDs were significantly more likely to have HBV and HCV infection. Rates of nucleic acid testing have reached nearly 100%.

**What are the implications for public health practice?**

Identification of HBV, HCV, and HIV risk factors among organ donors is critical to mitigate transmission risk and ensure monitoring and appropriate treatment of recipients for posttransplant infection. Nucleic acid testing has substantially reduced the period of undetectable infection.

## Footnotes

* https://www.cdc.gov/nchs/data/databriefs/db294.pdf.

† https://www.cdc.gov/hepatitis/statistics/2016surveillance/pdfs/2016HepSurveillanceRpt.pdf.

§ HBV surface antigen and core antibody, anti-HCV antibody, HCV NAT, and anti-HIV antibody testing are required for all donors; combined HIV antigen/antibody or HIV NAT are additionally required for IRDs. HBV NAT is not required for SRDs or IRDs.

¶ HBV: HBsAg, anti-HBc, and NAT; HCV: anti-HCV and NAT; HIV: anti-HIV, Ag/Ab and/or NAT.

**
https://optn.transplant.hrsa.gov/resources/guidance/phs-guideline-for-reducing-human-immunodeficiency-virus-hiv-hepatitis-b-virus-hbv-and-hepatitis-c-virus-hcv-through-organ-transplantation-frequently-asked-questions-2013/ .

††
https://optn.transplant.hrsa.gov/governance/public-comment/changes-to-hope-act-open-variance/ .
